# Evidence of nonverbal communication between nurses and older adults: a scoping review

**DOI:** 10.1186/s12912-020-00443-9

**Published:** 2020-06-16

**Authors:** Esther L. Wanko Keutchafo, Jane Kerr, Mary Ann Jarvis

**Affiliations:** Discipline of Nursing, School of Nursing and Public Health, University of KwaZulu-Natal, 71 Manor Drive, Manor Gardens, Durban, 4001 South Africa

**Keywords:** Nonverbal communication, Nurses, Older adults

## Abstract

**Background:**

Communication is an integral part of life and of nurse-patient relationships. Effective communication with patients can improve the quality of care. However, the specific communication needs of older adults can render communication between them and nurses as less effective with negative outcomes.

**Methods:**

This scoping review aims at describing the type of nonverbal communication used by nurses to communicate with older adults. It also describes the older adults’ perceptions of nurses’ nonverbal communication behaviors. It followed (Int J Soc Res 8: 19-32, 2005) framework. Grey literature and 11 databases were systematically searched for studies published in English and French, using search terms synonymous with nonverbal communication between nurses and older adults for the period 2000 to 2019.

**Results:**

The search revealed limited published research addressing nonverbal communication between older adults and nurses. The studies eligible for quality assessment were found to be of high quality. Twenty-two studies were included and highlighted haptics, kinesics, proxemics, and vocalics as most frequently used by nurses when communicating with older adults; while studies showed limited use of artefacts and chronemics. There was no mention of nurses’ use of silence as a nonverbal communication strategy. Additionally, there were both older adults’ positive and negative responses to nurses’ nonverbal communication behaviors.

**Conclusion:**

Nurses should be self-aware of their nonverbal communication behaviors with older adults as well as the way in which the meanings of the messages might be misinterpreted. In addition, nurses should identify their own style of nonverbal communication and understand its modification as necessary in accordance with patient’s needs.

## Background

Communication is a multi-dimensional, multi-factorial phenomenon and a dynamic, complex process, closely related to the environment in which an individual’s experiences are shared [1]. Regardless of age, without communication, people would not be able to make their concerns known or make sense of what is happening to them [2]. Communication links each and every person to their environment [3], and it is an essential aspect of people’s lives [4]. In healthcare settings, communication is essential in establishing nurse-patient relationships which contribute to meaningful engagement with patients, and the fulfilment of their care and social needs [5]. Effective communication is a crucial aspect of nursing care and nurse-patient relationships [6–8]. In healthcare encounters with older adults, communication is important, in particular to understand each person’s needs and to support health and well-being [9]. However, older adults may experience hearing deficits, changes in attention and coding of the information [10], and these communicative disabilities may restrict their interaction, participation and effective communication [11].

Communication occurs through verbal or nonverbal modalities [12, 13]. Nonverbal communication (NVC) is defined as a variety of communicative behaviors that do not carry linguistic content [14] and are the messages transmitted without using any words [15, 16]. NVC can act as a counter measure or an adjunct to verbal messages, in that it is more reliable if there is inconsistency between verbal and nonverbal messages [17]. Therefore, it is important that there is congruence between nonverbal and verbal messages [18], with research showing that patients are particularly alert to nurses and nurse-aids nonverbal behaviors [17, 19–21], especially when they are anxious and feel uncertain [14]. Despite the value of communication, it has been shown that healthcare workers spent very little time communicating with patients not satisfied with the information they received and how it was communicated [22]. Though verbal communication behaviors of healthcare providers have been extensively studied, their NVC behaviors have received less attention [17].

Scholars have varied in their estimations of the proportion of NVC in communication, with estimates as high as 93% [23], with other estimates of 60 to 90% [24]. Moreover, scholars have described different modalities of NVC, including artefacts (presence of physical and environmental objects), chronemics (use and perception of time), haptics (use of touch), kinesics (form of movement of the body), physical appearance (body type and clothing), proxemics (use of space and distance), vocalics (aspects of the voice), and silences [23, 25–27].

Concern needs to be directed on NVC and its different modalities as critical contributors to high quality care which plays a significant role in demonstrating respect for patients, fostering empathy and trusting provider-patient relationships [24]. A significant relationship exists between patient’s perceptions of empathy and eye contact and social touch [28], with touch, and gestures described as communication facilitators [27]. Nurses' positive facial expressions demonstrate signs of bonding, respect and affection towards older patients [29] while voice tones have contributed decisively to the success of interactions with older adults [30]. On the other hand, limited time has been reported by patients to have a negative impact on communication [31, 32], demonstrated in gestures of irritability which have caused embarrassment in older patients [29], and speaking fast has been a further communication barrier between nurses and patients [32]. The present review suggests the importance of understanding NVC between nurses and older adults, and underscores the need for focused research to address the gap in the knowledge of communication in geriatric care. The primary aim of the study was to identify the type of NVC strategies used by nurses to communicate with older adults in both acute care settings and long-term care settings.

## Methods

In order to map evidence-based knowledge and gaps [33–35] related to NVC between nurses and older adults, a systematic scoping review was conducted. Scoping reviews are useful to map the existing literature around a particular topic by charting findings and identifying research gaps [36], especially when the topic is complex or poorly reviewed [37]. A scoping review was chosen over a systematic review because the purpose of the study was to identify knowledge gaps related to nonverbal communication, as opposed to confirming or refuting the basis of current practice against relevant evidence [38]. The study adopted the framework proposed by Arksey and O’Malley [36] and further refined by Levac et al. [39]. The Preferred Reporting Items for Systematic reviews and Meta-Analyses extension for Scoping Reviews (PRISMA-ScR) Checklist [34] was followed for this review (Additional file 1).

### Research questions

The main question for this review was: What is the evidence of NVC between nurses and older adults? The sub questions were: (i) What are the different modalities of NVC used in geriatric nursing care? (ii) What are the functions of using the different NVC modalities? (iii) How do older persons respond to different NVC modalities?

### Eligibility criteria

The JBI framework of Population, Concept, Context (PCC) was used to determine the eligibility of the research question for this review (Table 1).

**Table 1:** PCC framework used to determine the eligibility of the research question

Criteria	Inclusion	Exclusion
Population	Professional nurses, registered nurses, enrolled nurses, nurse aides Nursing students	Nurses working in community settings All other healthcare workers Informal geriatric care givers
Concept	Nonverbal communication strategies and interpreted meaning between nurses and older adults (≥60 years)	Verbal communication between nurses and older adults Nonverbal communication strategies of older adults Nonverbal communication with nurses and older adults with communication impairments or disorders or dementia.
Context	Acute settings, nursing homes, long-term care	Acute hospital settings End-of-life / Terminal care unit; Psychiatric / mental health care unit; Communities

#### Population

Nurses including nursing students were considered in addition to qualified nurses and nurse aides because they are the largest population of healthcare workers [40].

#### Concept

The focus was NVC between nurses and older adults (≥60 years). For the purpose of this review, the United Nations cut-off of 60 years and older referring to the older adult population in Africa [41] was considered; yet, most Upper Income Countries have accepted the chronological age of 65 years and older, the age of retirement, as a definition of an older adult [42]. Socio-economic and disease reasons suggest that 65 years is not readily applicable to the African context [43].

Older adults with dementia were excluded although they are able to send and receive nonverbal information [39]. Dementia care combines comorbidities, cognitive and functional decline; leading to complex needs and ever-increasing difficulty for the patient in articulation [44], which is viewed as a challenging form of care.

#### Context

Acute settings and nursing homes were included into the context. In nursing homes, care is usually carried out by nursing staff with different levels of education and training [45]. Furthermore, community settings were excluded from the context because hospitalization is potentially stressful and involves unpleasant experiences for patients and their families [1], and thus offers a greater opportunity to identify the phenomenon under discussion.

### Search strategy

The search terms for this review originated from indexed subject headings, keywords of relevant studies, that recurred repetitively, and the Medical Subject Headings (MeSH) terms. The term ‘nonverbal communication’ was used as a starting point to develop a search string and identified other keywords to refer to NVC. The string/Boolean search terms for this review included: Participants (“nurses” OR “registered nurse” OR “professional nurses” OR “students nurses” OR “nurse aides”) AND Concept (“nonverbal communication” OR “kinesics” OR “proxemics” OR “artefacts” OR “chronemics” OR “haptics” OR “vocalics” OR “physical appearance” OR “active listening” OR “silences”) AND Context (“old people” OR “elder” OR “elderly” OR “older people” OR “aged” OR “geriatrics”).

### Database searching

A range of sources were used to ensure a comprehensive coverage of the literature. An initial search was conducted in August 2017, repeated and finalized in November 2019 The search made use of the following databases: Pubmed, Science Direct, Sabinet, Academic search complete, CINAHL with Full Text, Education Source, Health Source- Consumer Edition, Health Source: Nursing/Academic Edition, and MEDLINE. Google Scholar and Open Grey engines were also used to source relevant literature. Additionally, the reference lists of the included studies were used to search for additional studies. Only studies written in either English or French were retrieved.

Evidence of nurses’ NVC strategies while communicating with older adults, conducted in acute settings, and published in English or in French between 2000 and 2019 were included. Quantitative, qualitative, mixed-methods primary research studies, and reviews published in peer-reviewed journals, and grey literature that addressed the research question such as book chapters, thesis and reports were included. Evidence on communication with older adults suffering from communication impairment or dementia, in psychiatric units or communities, published in languages other than English or French were excluded. Evidence published before 2000 were excluded.

### Study selection

The titles were reviewed against the eligibility criteria by EW. This initial search was monitored, exported into EndNote X9 reference manager, for abstract and full text screening by EW. The duplicated studies were deleted, followed by independent reviewing of the abstracts by EW and JK. Studies deemed ‘unclear’ were advanced to the subsequent screening stage. Assistance from the study university library services was requested when full texts could not be retrieved from the databases and five full texts were provided. Full text of 75 eligible studies were independently filtered by EW and JK using Google forms. Additionally, a search of the reference list of all identified reports and studies for additional studies was performed by EW. MAJ pronounced on the discrepancies that occurred during the abstract screening and the full text screening until a consensus was reached.

### Data extraction

Information relevant to the aim of this study were extracted independently by EW and JK. A data extraction form was developed electronically using Google forms. Extracted data included bibliographic details, country and setting, aim/objective, study design, targeted population, nurses’ nonverbal strategies used while communicating with older adults, older adults’ interpretation of nurses’ nonverbal behaviors, and relevant outcomes of interest. Discussions between EK and JK refined the table of information extracted.

### Quality appraisal

The Mixed Methods Appraisal Tool (MMAT), version 2018 [46] was independently used by EW and JK to critically appraise the quality of the included primary studies. Discussion was used to resolve discrepancies. The MMAT allowed for assessment of the appropriateness of the aim of the study, adequacy and methodology, study design, participant recruitment, data collection, data analysis, and the presented findings [46]. The quality of studies was graded with a quality score ranging from ≤ 50% as low quality, 51–75% considered as an average quality, and 76–100% considered as high quality (Table 3).

### Collating and summarizing the data

Content thematic analysis approach [64] was employed to extract relevant data that answered the study questions. The results of the included studies were summarized, manually coded, and presented using a narrative approach. The nurses’ NVC behaviors were categorized under nine items namely (i) artefacts; (ii) chronemics; (iii) haptics; (iv) kinesics; (v) proxemics; (vi) vocalics; (vii) physical appearance; (viii) active listening; and (ix) silence.

## Results

**Fig. 1:**
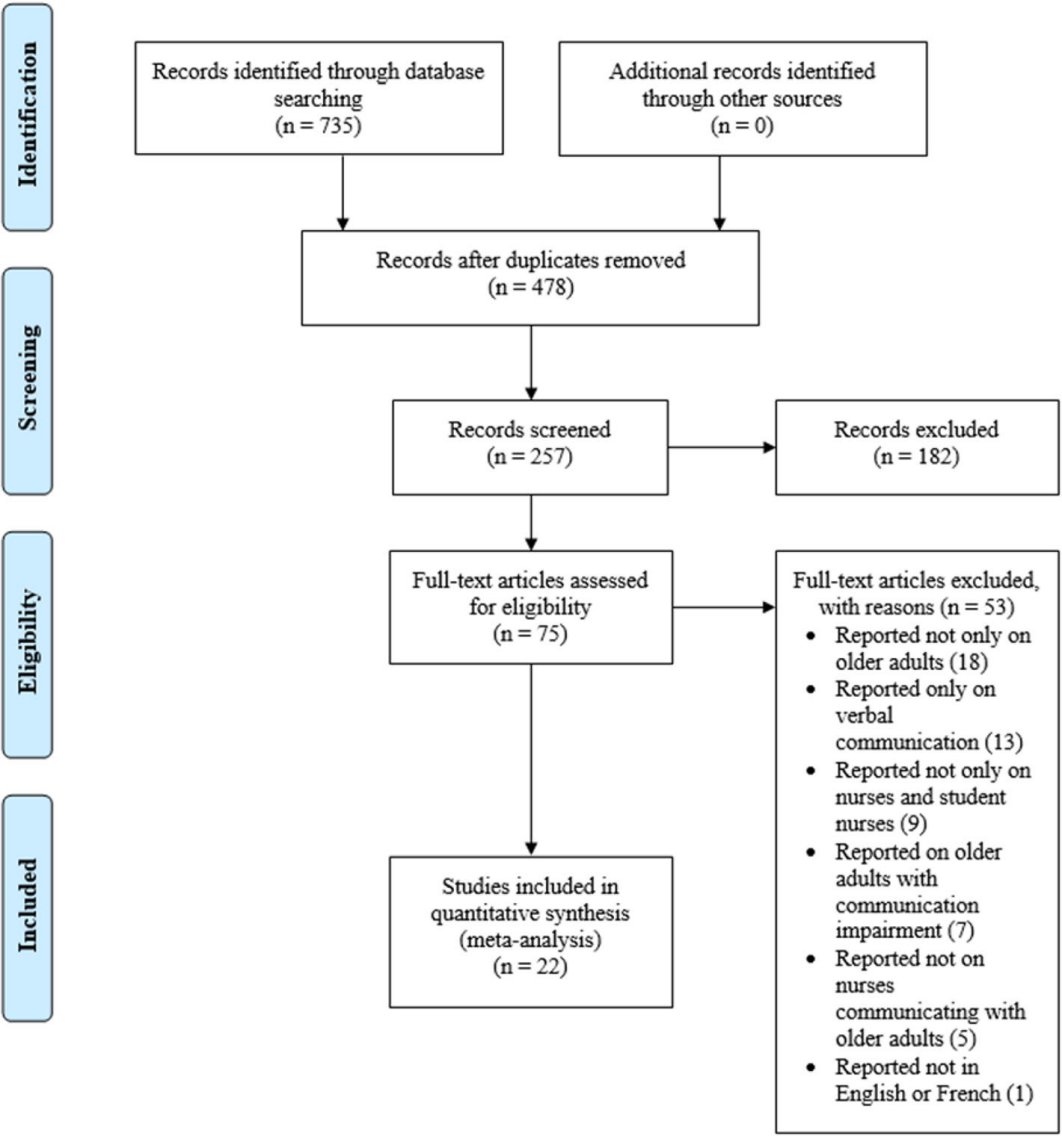
PRISMA 2009 Flow Diagram

Two hundred and fifty-seven (257) studies met the eligibility criteria following the deletion of 478 duplicates from the 735 studies identified at the title screening stage (Fig. 1).

### Characteristics of included studies

Tables 2 and 3 summarize the characteristics of the 22 included studies. All included studies were published in English and no eligible French studies were identified.

**Table 2:** Characteristics of the included studies (1)

Author(s) and year	Country	Setting	Design	Sample sizes	Quality appraisal
Johnsson et al. 2018 [47]	Sweden	Wards in a department of medicine for older people	Qualitative: observations, field conversations, and semi-structured interviews	40 nurses and 40 older adults	100%
Freitas 2016 [30]	Brazil	Family health unit	Qualitative: video recording	32 nurses and 32 older adults	100%
Small et al. 2015 [48]	Canada	Long term care	Qualitative: observation (video recording)s	27 staff and 27 older adults	100%
Freitas 2014 [49]	Brazil	Family health unit	Qualitative: video recording	32 nurses and 32 older adults	100%
Levy-Storms et al. 2011 [51]	USA	Nursing home	Qualitative: focus groups	17 nurse aides and 15 older adults	100%
Medvene and Lann-Wolcott 2010 [19]	USA	Assisted living facility and nursing home	Qualitative: individual interviews	16 nurse aides	100%
Backhaus 2009 [52]	Japan	Nursing home	Qualitative: observations	100 nurses and 57 older adults	100%
Gilbert and Hayes 2009 [53]	USA	Nurse practitioners’ offices	Mixed: observations (video recordings), survey	31 nurse practitioners and 155 older adults	100%
Sorensen 2009 [54]	the Balkans	Nursing home and rehabilitation unit	Qualitative: nursing students’ logs	10 third-year nursing students	100%
Williams and Warren 2009 [55]	USA	Assisted living facility	Qualitative: interviews and fieldwork	11 nursing assistants and 39 older adults	100%
Carpiac-Claver and Levy-Storms 2007 [57]	USA	Nursing homes and assisted living facilities in USA	Qualitative: observations (video recordings)	17 nurse aides and 17 older adults	100%
Kaakinen et al. 2001 [65]	USA	Care facilities, clinics, and private practice	Qualitative: one focus group and in-depth interviews	12 nurse practitioners	100%
Jonas 2006 [58]	Canada	Long term care	Qualitative: semi-structured interviews	19 older adults	100%
Tuohy 2003 [60]	Ireland	Continuing care unit	Qualitative: participant observations and eight semi-structured interviews	8 s year diploma nursing students	100%
Butts 2001 [62]	USA	Two nursing homes	Quantitative: randomized control trial	72 older adults	100%
Park and Song 2005 [59]	Korea	Medical, surgical, and ophthalmology units	Quantitative: survey	136 nurses and 100 older adults	80%
Daly 2017 [4]	Ireland	Not reported	Grey: Continuous Professional Development	N/A	N/A
Williams 2013 [50]	USA	Literature	Review	N/A	N/A
Calcagno 2008 [56]	USA	Long-term care	Grey: Continuous Professional Development	N/A	N/A
Linda 2002 [3]	UK	Not reported	Grey: Continuous Professional Development	N/A	N/A
Bush 2001 [61]	Germany	Not reported	Grey: author’ s reflection	N/A	N/A
Babikian 2000 [63]	USA	Long term care	Grey: authors’ reflection	N/A	N/A

**Table 3:** Characteristics of the included studies (2)

	Author(s) and year	Objective	Outcomes reported	Conclusions
2018	Johnsson et al. 2018 [47]	To describe how nurses communicate with older patients and their relatives in a department of medicine for older people in western Sweden	Nurses’ nonverbal communication strategies: standing position, eye gaze, speaking faster, speaking louder, speaking with a friendly tone, kneeling down, closing the door, smiling, facial expressions, smiling	Proxemics, kinesics, vocalics
2017	Daly 2017 [4]	To explore communication between nurses and older adults, with an emphasis on promoting effective communication in practice	Nurses’ nonverbal communication strategies: considering the environment, using touch appropriately, positioning oneself at the same level, active and compassionate listening	Artefacts, haptics, proxemics, active listening
2016	Freitas 2016 [30]	To assess proxemics communication between nurse and elderly in nursing consultation	Nurses’ nonverbal communication strategies: posture-Sex, sociofugo-sociopeto axis, distance evaluation, kinaesthetic, contact behaviour, visual code, thermal code, olfactory code, voice Volume	kinesics, vocalics, haptics, proxemics, artefacts
2015	Small et al. 2015 [48]	To explore the nature of communication between care staff and residents when they do not share the same language and ethno cultural backgrounds	Nurses’ nonverbal communication strategies: pointing, touching, eye gazing, smiling, sitting next, head nodding, playful gestures	kinesics, proxemics, haptics
2014	Freitas 2014 [49]	To analyse the performance of nurses in nursing consultation for the elderly based on the theoretical framework of Hall	Nurses’ nonverbal communication strategies: posture-sex, sociofugo-Sociopeto axis, distance evaluation, kinaesthetic, contact behaviour, visual code, thermal code, olfactory code, voice Volume	kinesics, vocalics, haptics, proxemics
2013	Williams 2013 [50]	To review evidence-based strategies for effective communication with older adults across long-term care settings	Nurses’ nonverbal communication strategies: eye contact, facial expressions, singing, humming, touching. Patients’ responses of nurses’ nonverbal communication strategies: dominance, disinterest	kinesics, haptics negative responses
2011	Levy-Storms et al. 2011 [51]	To characterise the meaning of and experiences with individualized care from the perspectives of both nursing aides and nursing-home residents	Nurses’ nonverbal communication strategies: listening, touching the shoulder Patients’ responses of nurses’ nonverbal communication strategies: respect, favouritism	haptics, active listening Mixed responses
2010	Medvene and Lann-Wolcott 2010 [19]	To identify the communication behaviours and strategies used by socially skilled geriatric nurse aides working with residents in long term care facilities	Nurses’ nonverbal communication strategies: touching, smiling, spending time with, observing body posture;	haptics, kinesics, chronemics
2009	Backhaus 2009 [52]	To examine the special nature of communication between residents and staff in a Japanese elderly care institution by taking a cross-cultural perspective	Nurses’ nonverbal communication strategies: kiss, hand shake, military tone	haptics, vocalics
2009	Gilbert and Hayes 2009 [53]	To examine contributions of older patients’ and nurse practioners’ characteristics and the content and relationship components of their communication to patients’ proximal outcomes and longer-term outcomes, and contributions of proximal outcomes to longer-term outcomes	Nurses’ nonverbal communication strategies: gaze, nod or shake of the head, eyebrow movement, smile, touch	kinesics, haptics
2009	Sorensen 2009 [54]	To demonstrate and discuss how personal competence, with emphasis on communication and empathy, can be developed by nursing students through international clinical practice	Nurses’ nonverbal communication strategies: body contact, pointing, nodding, smiling, laughing, active listening, voice pitch, thumbs up,	kinesics, vocalics, active listening, haptics
2009	Williams and Warren 2009 [55]	To explore how communication affects issues relating to residents maintaining cognitive and physical functioning so that they are able to remain in residence	Nurses’ nonverbal communication strategies: talk louder. Patients’ responses of nurses’ nonverbal communication strategies: rudeness; disinterest in; disdain for; perceived hypocrisy; threats to noncompliance; infantilization of residents; adultification of residents;	Vocalics Negative responses
2008	Calcagno 2008 [56]	To provide pointers to help clinicians listen to the needs and concerns of their clients	Nurses’ nonverbal communication strategies: greeting with a smile and handshake, sitting face-to-face, leaning forward, sitting close enough, listening, having an open posture	active listening, kinesics, proxemics
2007	Carpiac-Claver and Levy-Storms 2007 [57]	To identify types and examples of nurse aide-initiated communication with long-term care residents during mealtime assistance in the context of residents’ responses	Nurses’ nonverbal communication strategies: smiling, touching, laughing, singing, eye gazing, leaning forward, nodding, shaking hands, high pitch, soft tone	kinesics, haptics, vocalics
2001	Kaakinen et al. 2001 [65]	To describe communication between nurse practitioners and elderly clients	Nurses’ nonverbal communication strategies: touch, time, flyers, listening, drawings, pamphlets, written instructions; books; education files	artefacts, chronemics, haptics, active listening
2006	Jonas 2006 [58]	To explore the experience of being listened to for older adults living in long-term care facilities	Patients’ responses of nurses’ nonverbal communication strategies: nurturing contentment, vital genuine connections, respect and benefit	Active listening Positive responses
2005	Park and Song 2005 [59]	To determine and compare the communication barriers perceived by older inpatients and nurses caring for them, with the aim of identifying the disparities between the perceptions of the two parties	Nurses’ nonverbal communication strategies: speaking far away, without eye contact, with mask on, too loudly, too fast. Patients’ responses of nurses’ nonverbal communication strategies: working without a sincere attitude, being unfriendly, showing no respect	proxemics, kinesics, artefacts, vocalics negative responses
2003	Tuohy 2003 [60]	To ascertain how student nurses communicate with older people	Nurses’ nonverbal communication strategies: talking louder and slower, eye contact, facial expressions, appropriate touch	vocalics, kinesics, haptics
2002	Linda 2002 [3]	To explore the skills that are required for effective communication with older people	Nurses’ nonverbal communication strategies: body movements, postures, gestures, touch, proximity, pace of approach, eye contact, demeaning tone, speaking too quickly	kinesics, vocalics, haptics, proxemics
2001	Bush 2001 [61]	Author’s reflection on active listening	Nurses’ nonverbal communication strategies: leaning over, holding hand, active listening, eye contact, spending more time, notes, learning tools, posture, physical proximity	haptics, kinesics, active listening, chronemic, artefacts, proxemics
2001	Butts 2001 [62]	To examine whether comfort touch improved the perceptions of self-esteem, well-being and social processes, health status, life satisfaction and self-actualization, and faith or belief and self-responsibility	Patients’ responses to nurses’ nonverbal communication strategies: improved perception of self-esteem, well-being, social processes, health status, life satisfaction, self-actualisation, and faith or belief	Haptics Positive responses
2000	Babikian 2000 [63]	Author’s reflection on her encounter with an old person	Nurses’ nonverbal communication strategies: holding of hand, sitting next to	proxemics, haptics

### Study designs

Diverse research methods were employed within the 22 included studies. Thirteen studies were qualitative studies using individual interviews [19, 47, 55, 58, 60, 65], focus groups [51, 65], participant observations including video recordings [30, 47–49, 52, 57, 60], and participant logs [54]. There were one survey [59], one randomized controlled trial study [62] as well as one mixed methods study [53]. The other studies were a review [50] and two reflections [61, 63]. Three studies were related to continuous professional development [3, 4, 56].

### Quality of evidence

Of the 22 included studies, 16 studies underwent methodological quality assessment using the MMAT version 2018 [46]. The remaining six [3, 4, 50, 56, 61, 63] were excluded from the quality appraisal because they were not primary studies. The 16 studies which underwent methodological quality assessment showed high methodological quality and scored between 80 and 100%. Of these studies, 15 studies [19, 30, 47–49, 51–55, 57, 58, 60, 62, 65] scored 100%, and one [59] scored 80%.

### Study results

Three outcomes were reported in the studies: the NVC behaviors of nurses, the functions of those behaviors and the responses of older adults to the NVC behaviors.

### Nurses’ NVC behaviors and their functions

Of the 22 included studies, 20 reported on nurses’ NVC behaviors including haptics, kinesics, proxemics, vocalics, active listening, artefacts, and chronemics. There was no mention of physical appearance nor silences in all the included studies.

#### Haptics

Haptics were reported in 17 studies [3, 4, 19, 30, 48–54, 57, 60–63, 65] of which 12 studies, which underwent quality appraisal, were of high quality. Haptics were identified when nurses shook hands with older adults, held their hands, stroked or touched their hands. Nurses also kissed older adults, hugged them or gave them a pat on the shoulder.

In a study aiming at examining the special nature of communication between residents and staff in a Japanese elderly care institution, haptics were referred to as a handshake given by a member of staff against one older adults will [52]. This type of touch was used in a joking manner in Japan, where handshakes are uncommon, but was imposed on the older adult who did not appreciate it [52]. In another study conducted on types and examples of nurse aides-initiated communication with long-term care residents during mealtime assistance, haptics referred to a handshake when staff praised the older adults for eating all their food or to a touch on the arm for raising attention [57]. Stroking older adults’ hands were reported to be a means of conveying attention or affection while holding one older adult’s head back was used by a nurse to appease a negative response from the older adult in a study exploring the nature of communication between care staff and residents who did not share the same languages and ethno-cultural backgrounds [48].

Hugs were mentioned as a deliberate communication strategy used by a nurse practitioner to meet the unique needs of older adults in a study aimed at describing communication between nurse practitioners and older adults [65]. Hugs were also reported by nurses as a conscious NVC strategy specific to each older adult to establish rapport and prevent communication breakdowns between nurses and older adults [48]. An example was demonstrated through staff rubbing the sleepy older adult under the chin as a form of stimulus [48]. Additionally, a pat on the shoulder was mentioned as a caring gesture in a study aimed at characterizing the meaning of and experiences with individualized care from the perspectives of both nursing aides and nursing home residents [51]. However, a kiss on an older adult male’s forehead was described as inappropriate conduct [52].

#### Kinesics

Kinesics was reported in 14 studies [19, 30, 48–50, 53, 54, 56, 57, 59–61] of which 8 studies, which underwent quality appraisal, were of high quality. Nursing students developing personal competence in international clinical practice, used pointing and thumbs up, as movements of the hands, to communicate nonverbally when words were in short supply [54]. Further, a Swedish study described nurses’ use of pointing to communicate with older adults and their relatives in a department of medicine for older adults [47].

Kinesics also referred to as movements of the head, included facial expressions, movements of the eyes, and head nods. Student nurses’ use of facial expressions and eye contact were described as components of effective communication with older adults [60]. While facial expressions such as a smile and laugher were reported to both quickly and amicably resolve disagreements between staff and older adults, smiles were seen as enhancers of the communication in a study where staff occasionally engaged in smiling with older adults [48]. A nurses’ smile was also seen as a way to either convey the message [54], to initiate communication [57] or an attempt to create a positive atmosphere during the meeting with older adults [47].

Student nurses described head nodding as a means to convey their message nonverbally when communicating with older adults [54]. Nodding was also used to convey communicative intent nonverbally, to indicate acceptance or rejection of staff’s actions [48], and to address or to praise the older adult [57]. Additionally, nodding was used by nurses to show that they had understood what older adults and their relatives had said [47].

Eye gaze was seen as nurses’ willingness to be engaged in conversation in a review on evidence-based strategies for effective communication with older adults across long-term care settings [50]. Eye gaze was also used to gain older adults’ attention, or as means to both connect relationally and instrumentally [48]. Additionally, eye gazing was used to gain older adults’ attention, when the nature of communication between care staff and residents using different languages and having ethno-cultural backgrounds was explored [48]. Eye contact was suggested as advice to effectively communicate with older adults [3], or a means of improving communication skills [61]. However, Visual Code factor was among the factors that received the lowest scores in a study analyzing the performance of nurses in nursing consultation for the older adults based on the theoretical framework of Hall [49]. The low score was justified by the unpreparedness of nurses about the aging process [49].

Movements of the body included leaning over older adults to assess their progress [61] or to check on them in a study conducted on types and examples of nurse aide-initiated communication with long-term care residents during mealtime assistance [57]. Additionally, leaning forward was a means to indicate the nurses’ eagerness and readiness to listen to the older adults’ stories, in a study providing pointers to help clinicians listen to the needs and concerns of older adults [56].

#### Proxemics

Proxemics, defined as the social meaning of space and interactive field, which determines how relationships occur [115] were reported in 10 studies [3, 4, 30, 47, 49, 56, 59, 61, 63], and included physical proximity and physical distance. Of these studies, eight were of high quality based on the MMAT assessment.

Speaking far away was mentioned as a nurse-related communication barrier perceived by both older adults and nurses [59]. Additionally, a Swedish study noted that nurses remained standing while using a medical voice to communicate with older adults, [47]. In contrast, nurses positioning themselves at the same level as older adults was a strategy to support their communication with older adults [4]. Sitting next to older adults was part of the playful gestures nurses engaged in, in a study which explored the nature of communication between care staff and residents with different languages and ethno-cultural backgrounds [48]. Likewise, pointers to help clinicians listen to the needs and concerns of older persons included physical presence to enhance the ability to listen and show interest [46], sitting by the older adult’s side to hold their hand [63], sitting face to face to indicate presence and the readiness to listen [56]. On the contrary to the literature supporting engagement on the same plane, kneeling down was also used by nurses to make eye contact with older adults and seen in the instance of planning a good home return [47].

#### Vocalics

Vocalics were reported in nine studies [30, 47, 49, 52, 54, 55, 57, 59, 60] where they described different aspects of the voice tone and sense of calm. All the eight studies that underwent quality appraisal were of high quality.

A military tone with endearment used to address an older adult, in a Japanese elderly care institution, was not appreciated even though used in jest [52]. Conversely, although to no avail, a soft tone was used by a nurse to encourage an older adult to eat her food [57]. Additionally, speaking too quickly and in a demeaning tone were reported as barriers to effective communication [3]. Likewise, speaking too loudly and speaking too fast were nurse-related communication barriers as perceived by nurses and older adults [59]. Speaking faster and with a monotonous tone were reported when nurses used a medical voice to communicate with older adults as well as speaking louder and with great emphasis on selected words were reported when nurses used a power voice [47].

Conversely, speaking calmly contributed to create mutual trust in the student nurse-older adult relationships in a study demonstrating that communication and empathy can be developed by student nurses through clinical practice [54]. Speaking slower was a means for student nurses to be understood by older adults [60], and speaking with a friendly tone was used by nurses to increase the knowledge of older adults [47]. Additionally, the tone used by nurses favored communication with older adults and made possible the understanding of what was being expressed in a study aimed at assessing proxemics communication between nurse and elderly in nursing consultation [30].

#### Listening

Listening was reported in seven studies [4, 51, 54, 56, 58, 61, 65] of which four studies were eligible for quality appraisal and scored 100% on the MMAT assessment.

In one instance, listening was reported as a means to help nurses assess older adults’ physical condition more effectively [61]. Active listening coupled with compassionate listening was a strategy to support nurses’ communication with older adults [4], and proven to be helpful [65]. Emphatic, non-judgmental listening, while being aware of the body language of the older adults, provided pointers to help nurses listen to the needs and concerns of their clients [56].

Actively listening to older adults’ verbal and NVC behaviors was seen as leading to individualized care and a sign of respect to older adults in a study characterizing the meaning of and experiences with individualized care from the perspectives of both nursing aides and nursing-home residents [51]. Nursing students, associated active listening in relation to NVC as an empathic response and an open - minded attitude [54].

#### Artefacts

Artefacts were reported in five of the 22 included studies [4, 30, 59, 61, 65] of which three studies eligible for quality appraisal were of high quality.

Artefacts were communication supports and aids that can support nurses’ communication with older adults [4]. Artefacts included notes and hands-on learning tools as strategies to improve communication [61] as well as flyers, pamphlets, written instructions, books and education files [65].

When promoting effective communication in practice, it was advised that nurses should be mindful of the physical environment that can affect interactions between them and older adults [4]. The results show that nurses closed the door of an older adult’s room to avoid any disturbance of the communication exchange in Sweden [47], while nurses performed their service with the door opened and allowed excessive entry of others into the room while consulting older adults in Brazil [30]. Nurses should guarantee privacy and should avoid speaking while wearing a mask as it is considered as an impediment to effective communication [59].

#### Chronemics

There was lesser reporting of chronemics and NVC, described in only three studies [19, 61, 65] and only one study eligible for quality assessment was of good quality [19].

In a study aimed at identifying the communication behaviors and strategies used by socially skilled geriatric nurse aides working with residents in long term care facilities, spending time with older adults was described by the nurses as giving them positive regard, explained as being respectful, acknowledging and showing interest and approval [19]. In a reflection on active listening, spending more time with older patients was mentioned as a means to promote feelings of acceptance, and exercising patience as the most challenging part of the communication process [61]. Likewise, time was found to positively affect nurse practitioners-older adults relationships [65].

### Old adults’ responses to nurses’ NVC behaviors

Six studies [50, 51, 55, 58, 59, 62] reported on the older adults’ responses to nurses’ NVC behaviors. The responses were either positive or negative.

#### Positive responses

Positive responses to nurses’ NVC behaviors were reported in three studies [51, 58, 62]. Comfort touch from nurses was shown to improve the perceptions of self-esteem, well-being, social processes, health status, life satisfaction, self-actualization, and faith or belief [62] while a pat on the shoulder was perceived as a sign of respect [51]. In a study exploring the experience of being listened to, for older adults living in long-term care facilities, results showed they expressed their satisfaction, gratification, and unburdening and described their relationships with the nurses who listened to them as being close like friends or family [58].

#### Negative responses

Negative responses to nurses’ NVC behaviors were reported in four studies [50, 51, 55, 59]. In a study aimed at exploring how communication affects issues relating to residents maintaining cognitive and physical functioning in order to remain in the residence, vocalics were perceived by the nurses as rudeness, disinterest, “infantilisation” and “adultification” [55]. In a study with the aim to determine and compare the communication barriers perceived by older adults and nurses caring for them, speaking far away, without eye contact, wearing a mask and too loud was perceived as being unfriendly, working without a sincere attitude, and showing no respect [59]. In a review of evidence-based strategies for effective communication with older adults across long-term care settings, touching their buttocks or looming over them were perceived by older adults as dominance, while glancing at their watch or down the hall was perceived as a sign of disinterest [50].

## Discussion

This systematic scoping review explored evidence on NVC between nurses and older adults, focusing on cognitively intact older adults with no mental illness nor communication impairment. A total of 22 studies were included. Haptics, kinesics, proxemics, and vocalics were the most frequently used NVC strategies by nurses when communicating with older adults, of which 15 scored 100% on MATT. This study’s findings further demonstrate a limited use of artefacts and chronemics as forms of NVC. Physical appearance regarding NVC was not mentioned in any of the included studies nor was silence. The results evidenced limited published research in the select topic and in particular for studies located in Asia and Africa, as well as for quantitative studies. Though the majority of studies were qualitative designs, which do not allow generalization of findings, the quality of the included studies ensures credibility.

The majority of the studies included in this review illustrate the different modalities of nurses’ NVC behaviors in geriatric nursing care. The most cited NVC behaviors were haptics perhaps because touch is an essential and often unavoidable part of nursing care [66]. Haptics or communication by touch [67] can include aggressive touch, accidental touch, playful touch, task related touch [68] or task-oriented touch, touch promoting physical comfort, and touch providing emotional containment [66, 68]. In the included studies, touch was used to joke, to praise, to get attention, to convey attention, to stimulate, and to show care. In one instance, touch was not appreciated by the older adult [52], which highlights that touch can lead to either positive or negative outcomes, depending on the nurses’ awareness and intention [69]. Touch can be a nursing tool [70], but nurses need to use touch appropriately, taking into consideration preferences and avoiding its imposition on older adults.

Kinesics are different from haptics in the sense that there is no contact with a person, and only movements of the hands, head, and the body are used. Kinesics were used when words were in short supply, to convey messages, to indicate acceptance or rejection by either party, to resolve disagreements amicably and with speed, to initiate communication, to get attention, and lastly to praise. Gesturing with a meaning of rejection or disapproval as well as abrupt gestures interrupt the exchange of messages [71], highlighting the need for nurses to ensure correct decoding of kinesics [71]. Also, it is important that nurses keep eye contact with older adults during interactions, keeping in mind that the permission of this contact may vary depending on culture [30].

Proxemics included personal space and territoriality [72] and included sitting next to, face-to-face, beside the person, kneeling, looming over, and speaking far away from the person. Proximity can therefore indicate presence, readiness to listen, and a sign of interest in the older adult. Distance can be seen as a barrier to effective communication with older adults. There should be a balance between distance and proximity, with nurses mindful of the often-invasive nature of nursing, and the need to create a therapeutic space where older adults’ privacy is not violated.

Vocalics are often associated with “elderspeak”, which in addition includes oversimplifying the language, speaking at a slow rate, loud, and with a demeaning tone [73]. In this study, vocalics included speaking with a military or a demeaning tone, speaking too fast or too loud, which led to negative outcomes while speaking calmly or slower led to positive outcomes. Conversely, speaking with a soft tone also led to a negative outcome [57]. In light of the importance of nurses developing self-awareness of the tone that they use to communicate, an opportunity exists for them to use audio recordings to reflect on their tone [3].

Physical appearance was not mentioned in any of the included studies; yet, the clothing worn in nursing is a form of NVC that frequently shapes people’s judgments about others, regardless of whether or not the perceptions are true [74]. Therefore, nurses should be aware that the way they present themselves through their uniforms might indirectly communicate something about the care they render.

Positive responses to nurses’ NVC behaviors included improved perceptions of self-esteem, well-being, health status, and faith as well as expressed satisfaction and gratification when being listened to by nurses. On the other hand, older adults viewed vocalics used by nurses as a sign of rudeness and disinterest, while nurses who used proxemics were perceived as being unfriendly, working without a sincere attitude, and showing no respect. In order to avoid negative responses from older adults, a level of trust between nurses and older adults needs to precede touch [75]. Though nurses–patients’ communication is influenced by conditions that arise in hospital settings, [76], nurses need to adjust their communication style to each situation and each patient [77].

### Implications for practice

Awareness of NVC will lead to a greater understanding of the messages exchanged [74]. When the essence of nursing care falls short, all other initiatives are more likely to fail as well [78], implying that if communication with older adults is hindered or tampered with, everything else nurses engage in is likely to fail. Nurses need to be self-aware of their NVC as well as the way in which the meanings of the messages might be misinterpreted, highlighting a need for interventions to aid nurses to interact and communicate holistically with older adults [79]. Additionally, when nurses are aware that older adults are not a homogenous group subject to general assumptions of care [4], communication barriers created by nurses create barriers [61] would be avoided.

### Implications for education

An emphasis should be placed on teaching effective communication to prepare future healthcare providers to minimize miscommunication, deliver safe, quality care, and contribute to anti-ageism measures. Also, the training of nurse on NVC will enable the establishment of bonds with older adults and culminate in effective care [49]. Preparation of the neophytes will ensure a sustainable, older-person centered and appropriately trained workforce as advocated by the WHO (2016) [43].

### Implications for research

This scoping review draws attention to the limited evidence, specific to NVC between nurses and older adults without mental illness, or communication impairment, indicating a gap in literature, in particular in Asian and African countries. In addition, this review highlights the need for further research to provide an African insight into NVC to answer the WHO call for more data to understand the needs and the status of older adults in Africa [80]. We further recommend a study to determine the impact of nurses’ NVC behaviors on older adults’ satisfaction and safety of care. Though time constraints can sometimes prevent nurses from providing the attentive communication older adults seek, it is important that nurses identify their own style of NVC and understand how to modify, when necessary, their interactions with patients, in particular older persons.

### Strengths and limitations

#### Strengths

This study is possibly the first scoping review to map evidence on NVC between nurses and older adults with neither mental illness nor communication impairment. This study demonstrated a substantial gap in the NVC literature to guide future research on older adults with no mental illness or communication impairment. The study’s methodology also allowed the inclusion of different study designs, and the identification of relevant studies methodically charting, and analyzing the outcomes.

#### Limitations

Despite the inclusion of MeSH terms, it is possible that research on NVC existed under different terminologies, which were not captured in this review. As only abstracts written in English and French were included, some relevant studies may have been missed. Several studies of NVC between nurses and older adults may have been reported only in contexts of mental illnesses or communication deficiencies, leading to their exclusion from this review. Additionally, studies on NVC between other healthcare workers and older adults have not been reviewed.

## Conclusions

This study explored evidence on NVC between nurses and older adults with no mental illness nor communication impairment. The results revealed that haptics, kinesics, proxemics, and vocalics were the most frequently used NVC strategies by nurses while there was a limited use of artefacts and chronemics as forms of NVC. Furthermore, physical appearance and silence were not mentioned in any of the 22 included studies. Nurses used NVC strategies to joke, to praise, to get or convey attention, to stimulate, to show care, to indicate acceptance or rejection, to resolve disagreements amicably, to initiate communication, to indicate presence, readiness to listen, and a sign of interest in the older adults. Lastly, older adults responded to nurses’ NVC behaviors either in a positive way or in a negative way.

## Supplementary information

**Additional file 1.**

**Additional file 2.**

## Data Availability

Data sharing is not applicable to this article.

## Abbreviations

MMAT: Mixed Methods Appraisal Tool
MeSH: Medical Subject Headings
NVC: Nonverbal Communication
PCC: Population, Concept, Context
PRISMA-ScR: Preferred Reporting Items for Systematic Reviews and Meta-Analyses extension for Scoping Reviews
WHO: World Health organization

## References

[1] Norouzinia R, Aghabarari M, Shiri M, Karimi M, Samami E. Communication barriers perceived by nurses and patients. Global J Health Sci. 2016;8(6):65-74. 10.5539/gjhs.v8n6p65 PMC4954910 26755475

[2] Casey A, Wallis A (2011). Effective communication: principle of nursing practice E. Nurs Stand.

[3] Linda M (2002). Effective communication with older people. Nurs Stand.

[4] Daly L. Effective communication with older adults. Nursing Standard (2014+). 2017;31(41):55-63. 10.7748/ns.2017.e10832 28589825

[5] Wiechula R, Conroy T, Kitson AL, Marshall RJ, Whitaker N, Rasmussen P. Umbrella review of the evidence: what factors influence the caring relationship between a nurse and patient? J Adv Nurs. 2016;72(4):723–34. 10.1111/jan.12862 26692520

[6] Kounenou K, Aikaterini K, Georgia K. Nurses’ communication skills: exploring their relationship with demographic variables and job satisfaction in a Greek sample. Procedia Soc Behav Sci. 2011;30(2011):2230–4.

[7] Martin A-M, O'Connor-Fenelon M, Lyons R (2010). Non-verbal communication between nurses and people with an intellectual disability: a review of the literature. J Intellect Disabil.

[8] Williams KN, Boyle DK, Herman RE, Coleman CK, Hummert ML (2012). Psychometric analysis of the emotional tone rating scale: a measure of person-centered communication. Clin Gerontol.

[9] Hafskjold L, Sundler AJ, Holmström IK, Sundling V, van Dulmen S, Eide H. A cross-sectional study on person-centred communication in the care of older people: the COMHOME study protocol. BMJ Open. 2015;5(4):1-9. 10.1136/bmjopen-2015-007864 PMC4401848 25877282

[10] Sanecka A. Social Barriers to Effective Communication in Old Age. J Educ Cult Soc. 2014;2014(2):144–53.

[11] Forsgren E, Skott C, Hartelius L, Saldert C. Communicative barriers and resources in nursing homes from the enrolled nurses’ perspective: a qualitative interview study. Int J Nurs Stud. 2016;54(2016):112–21. 10.1016/j.ijnurstu.2015.05.006 26087703

[12] Araújo MMT, Silva MJP. Estratégias de comunicação utilizadas por profissionais de saúde na atenção à pacientes sob cuidados paliativos. Rev Esc Enferm USP. 2012;46(3):626–32. 10.1590/s0080-62342012000300014 22773483

[13] Zani A, Marcon S, Tonete V, Parada C. Communicative process in the emergency department between nursing staff and patients: social representations. Online Braz J Nurs. 2014;13(2):139-49.

[14] Fernández EI. Verbal and nonverbal concomitants of rapport in health care encounters: implications for interpreters. J Specialized Transl. 2010;14(2010):216–28.

[15] McEwen A, Harris G. Commununication Skills Adult Nurses. Beckshire: University Press; 2010. Chapter 1, Communication: fundamental skills; p. 1-22.

[16] Stickley T (2011). From SOLER to SURETY for effective non-verbal communication. Nurse Educ Pract.

[17] Xu Y, Staples S, Shen JJ (2012). Nonverbal communication behaviors of internationally educated nurses and patient care. Res Theory Nurs Pract.

[18] Kourkouta L, Papathanasiou IV (2014). Communication in nursing practice. Mater Sociomed.

[19] Medvene LJ, Lann-Wolcott H (2010). An exploratory study of nurse aides’ communication behaviours: giving ‘positive regard’ as a strategy. Int J Older People Nursing.

[20] Liu JE, Mok E, Wong TJJCN (2006). Caring in nursing: investigating the meaning of caring from the perspective of cancer patients in Beijing, China 1. J Clin Nurs.

[21] Röndahl G, Innala S, Carlsson M (2006). Heterosexual assumptions in verbal and non-verbal communication in nursing. J Adv Nurs.

[22] Zarea K, Maghsoudi S, Dashtebozorgi B, Hghighizadeh MH, Javadi M. The impact of peplau's therapeutic communication model on anxiety and depression in patients candidate for coronary artery bypass. Clin Pract Epidemiol Mental Health. 2014;10(2014):159-65. 10.2174/1745017901410010159 PMC4262794 25505931

[23] Wold G (2013). Basic geriatric nursing - E-book.

[24] Lorié Á, Reinero DA, Phillips M, Zhang L, Riess H (2017). Culture and nonverbal expressions of empathy in clinical settings: a systematic review. Patient Educ Couns.

[25] Boggs K, Arnold E, Boggs K (2015). Variation in communication styles. Interpersonal Relationships: Professional Communication Skills for Nurses.

[26] Wittenberg-Lyles E, Goldsmith J, Ferrell B, Ragan S (2013). Communication in palliative nursing.

[27] Stanyon M, Griffiths A, Thomas S, Gordon A (2016). The facilitators of communication with people with dementia in a care setting: an interview study with healthcare workers. Age Ageing.

[28] Montague E, Chen P-y, Xu J, Chewning B, Barrett B (2013). Nonverbal interpersonal interactions in clinical encounters and patient perceptions of empathy. J Participat Med.

[29] de Almeida RT, Ciosak SI (2013). Communication between the elderly person and the family health team: is there integrality?. Rev Lat Am Enfermagem.

[30] Freitas FFQ, Mendes JMS, de Medeiros TM, da Costa TF, Fernandes MGM, Costa KNFM. Proxemic assessment of relations between nurse and elderly in nursing consultations. Int Arch Med. 2016;9(66):1-9.

[31] Chan E, Wong F, Cheung M, Lam W (2018). Patients’ perceptions of their experiences with nurse-patient communication in oncology settings: a focused ethnographic study. PLoS One.

[32] Jahromi M, Ramezanli S (2014). Evaluation of barriers contributing in the demonstration of an effective nurse-patient communication in educational hospitals of Jahrom. Global J Health Sci.

[33] Colquhoun HL, Levac D, O'Brien KK, Straus S, Tricco AC, Perrier L, Kastner M, Moher D (2014). Scoping reviews: time for clarity in definition, methods, and reporting. J Clin Epidemiol.

[34] Tricco AC, Lillie E, Zarin W, O'Brien KK, Colquhoun H, Levac D, Moher D, Peters MDJ, Horsley T, Weeks L (2018). PRISMA extension for scoping reviews (PRISMA-ScR): checklist and ExplanationThe PRISMA-ScR statement. Ann Intern Med.

[35] The Joanna Briggs Institute, Institute TJB (2015). Joanna Briggs institute reviewers’ manual: 2015 edition / supplement. Methodology for JBI Scoping Reviews.

[36] Arksey H, O'Malley L (2005). Scoping studies: towards a methodological framework. Int J Soc Res Methodol.

[37] Dijkers M (2015). What is a scoping review?. KT Update.

[38] Munn Z, Peters MDJ, Stern C, Tufanaru C, McArthur A, Aromataris E. Systematic review or scoping review? Guidance for authors when choosing between a systematic or scoping review approach. BMC Med Res Methodol. 2018;18(1):1-7. 10.1186/s12874-018-0611-x PMC6245623 30453902

[39] Levac D, Colquhoun H, O'Brien KK. Scoping studies: advancing the methodology. Implement Sci. 2010;5(1):1-9. 10.1186/1748-5908-5-69 PMC2954944 20854677

[40] Rush K (2017). Nurses’ attitudes towards older people care: an integrative review. J Clin Nurs.

[41] Kowal PR, Wolfson LJ, Dowd JE. Creating a minimum data set on ageing in sub-Saharan Africa. South Afr J Gerontol. 2000;9(2):18–23.

[42] Zverev Y (2013). Attitude towards older people among Malawian medical and nursing students. Educ Gerontol.

[43] World Health Organization. Health systems. 2016. Available from: http://www.who.int/topics/health_systems/en/. Accessed 17 Jan 2017.

[44] Evripidou M, Charalambous A, Middleton N, Papastavrou E (2019). Nurses’ knowledge and attitudes about dementia care: systematic literature review. Perspect Psychiatr Care.

[45] Bing-Jonsson PC, Hofoss D, Kirkevold M, Bjørk IT, Foss C (2016). Sufficient competence in community elderly care? Results from a competence measurement of nursing staff. BMC Nurs.

[46] Hong QN, Pluye P, Fàbregues S, Bartlett G, Boardman F, Cargo M, Dagenais P, GagnonM-P GF, Nicolau B, O'Cathain A (2018). Mixed methods appraisal tool (MMAT), version 2018. IC Canadian Intellectual Property Office.

[47] Johnsson A, Boman Å, Wagman P, Pennbrant S (2018). Voices used by nurses when communicating with patients and relatives in a department of medicine for older people—an ethnographic study. J Clin Nurs.

[48] Small J, Chan SM, Drance E, Globerman J, Hulko W, O’Connor D, Perry J, Stern L, Ho L (2015). Verbal and nonverbal indicators of quality of communication between care staff and residents in ethnoculturally and linguistically diverse long-term care settings. J Cross Cult Gerontol.

[49] Freitas FF, de Oliveira LJ, Bezerra Oliveira CD, Oliveira e Silva AC, Macêdo Silva J, Neyla de Freitas Macedo Costa K (2014). Consultation performance of nursing for the elderly: Analysis based on the theory of hall. J Nurs UFPE.

[50] Williams K (2013). Evidence-based strategies for communicating with older adults in long-term care. J Sci Commun.

[51] Levy-Storms L, Claver M, Gutierrez VF, Curry L (2011). Individualized care in practice: communication strategies of nursing aides and residents in nursing homes. J Appl Commun Res.

[52] Backhaus P (2009). Politeness in institutional elderly care in Japan: a cross-cultural comparison. J Politeness Res Lang Behav Cult.

[53] Gilbert DA, Hayes E (2009). Communication and outcomes of visits between older patients and nurse practitioners. Nurs Res.

[54] Sørensen AL. Developing personal competence in nursing students through international clinical practice: with emphasis on communication and empathy. J Intercult Commun. 2009;2009(19):1-7.

[55] Williams KN, Warren CAB (2009). Communication in assisted living. J Aging Stud.

[56] Calcagno KM (2008). Listen up … someone important is talking. Home Healthcare Now.

[57] Carpiac-Claver ML, Levy-Storms L (2007). In a manner of speaking: communication between nurse aides and older adults in long-term care settings. Health Commun.

[58] Jonas-Simpson C, Mitchell GJ, Fisher A, Jones G, Linscott J (2006). The experience of being listened to: a qualitative study of older adults in long-term care settings. J Gerontol Nurs.

[59] Park E-k, Song M. Communication barriers perceived by older patients and nurses. Int J Nurs Stud. 2005;42(2):159–66. 10.1016/j.ijnurstu.2004.06.006 15680614

[60] Tuohy D (2003). Student nurse-older person communication. Nurse Educ Today.

[61] Bush K. Do you really listen to patients? RN. 2001;64(3):35-7. 11288647

[62] Butts JB (2001). Outcomes of comfort touch in institutionalized elderly female residents. Geriatr Nurs.

[63] Babikian MY (2000). High touch. J Gerontol Nurs.

[64] Clarke V, Braun V, Terry GN, Liamputtong P (2019). H: Thematic analysis. Handbook of research methods in health and social sciences. edn.

[65] Kaakinen J, Shapiro E, Gayle BM (2001). Strategies for working with elderly clients: a qualitative analysis of elderly client/nurse practitioner communication. J Am Assoc Nurse Pract.

[66] Pedrazza M, Trifiletti E, Berlanda S, Minuzzo S, Motteran A (2015). Development and initial validation of the nurses’ comfort with touch scale. J Nurs Meas.

[67] Bobby CS. Haptic Communication-The Unspoken Dialogue. Lang India. 2014;14(4):546-55.

[68] Patterson A, Berg M. Exploring nonverbal communication through service learning. J Civic Commitment. 2014;22. Available from: http://ccncce.org/articles/exploring-nonverbal-communication-through-service-learning/.

[69] Pedrazza M, Berlanda S, Trifiletti E, Minuzzo S. Variables of individual difference and the experience of touch in nursing. West J Nurs Res. 2017;0(0):1-24. 10.1177/0193945917705621 28459179

[70] Airosa F, Falkenberg T, Öhlén G, Arman M (2016). Tactile massage as part of the caring act: a qualitative study in short-term emergency wards. J Holist Nurs.

[71] Borges P, Wicto J, Magalhães Moreira TM, Braz da Silva D, Oliveira Loureiro AM, de Meneses B, Viana A. Adult nursing-patient relationship: Integrative review oriented by the king interpersonal system. J Nurs UFPE. 2017;11(4):1769-78.

[72] Azevedo AL, Araújo STC, Pessoa Júnior JM, Silva J, Santos BTU, Bastos SSF. Communication of nursing students in listening to patients in a psychiatric hospital. Escola Anna Nery. 2017;21(3):1-6.

[73] Williams KN. Communication in elderly care: Cross-cultural perspectives. London: Continuum, 2011. Elderspeak in institutional care for older adults; p. 1–19. Available from: https://ir.uiowa.edu/nursing_pubs/1890.

[74] Sudirman I, Sidin I (2016). Does demography matter in nonverbal communication between physician and patient. Res J Bus Manag.

[75] Gillham D, De Bellis A, Xiao L, Willis E, Harrington A, Morey W, Jeffers L (2018). Using research evidence to inform staff learning needs in cross-cultural communication in aged care homes. Nurse Educ Today.

[76] Fakhr-Movahedi A, Rahnavard Z, Salsali M, Negarandeh R (2016). Exploring Nurse’s communicative role in nurse-patient relations: a qualitative study. J Caring Sci.

[77] Prip A, Pii KH, Møller KA, Nielsen DL, Thorne SE, Jarden MJEJON (2019). Observations of the communication practices between nurses and patients in an oncology outpatient clinic. J Oncol Nurs.

[78] Feo R, Kitson A (2016). Promoting patient-centred fundamental care in acute healthcare systems. Int J Nurs Stud.

[79] Deane WH, Fain J (2016). Incorporating Peplau’s theory of interpersonal relations to promote holistic communication between older adults and nursing students. J Holist Nurs.

[80] United Nations Department of Economic and Social Affairs Population Division (2016). Population facts (2010/2/E).

