# Supplementary material for: Evidence of nonverbal communication between nurses and older adults: a scoping review
Source: BMC Nurs. 2020 Jun 16;19:53. doi: 10.1186/s12912-020-00443-9 (PMC7298765; doi:10.1186/s12912-020-00443-9)
Supplement: Supplementary file 2 — Additional file 2. [file 12912_2020_443_MOESM2_ESM.docx]

**Table 4: Examples of nonverbal communication strategies**

| **NVC strategy** | **Definition** | **Examples** |
| --- | --- | --- |
| Haptics | Use of touch | Task-related touch  Affective touch (caress, kiss, holding hand, touching shoulder) |
| Kinesics | Forms of movement of the body | Movements of the head (facial expressions, eye gaze, smile)  Movements of the hands (finger pointing, hand shake)  Movement of the body (leaning forward or backward, dancing) |
| Proxemics |  | Physical distance (sitting next to, kneeling down)  Physical proximity (speaking far from, standing at the door) |
| Vocalics | Aspects of the voice | Soft tone, high pitch, commanding tone, demeaning tone, friendly tone, speaking fast |
| Chronemics | Use and perception of time | Spending time, rushing |
| Artefacts | Presence of physical and environmental objects | Pamphlets, flyers, bottles, noise |
| Physical appearance | Body type and clothing | Cleanliness of the uniform, hair style, makeup |
